# Quantifying a Learning Curve for Video Head Impulse Test: Pitfalls and Pearls

**DOI:** 10.3389/fneur.2020.615651

**Published:** 2021-01-22

**Authors:** Athanasia Korda, Thomas C. Sauter, Marco Domenico Caversaccio, Georgios Mantokoudis

**Affiliations:** ^1^Department of Otorhinolaryngology, Head and Neck Surgery, Inselspital, University Hospital Bern and University of Bern, Bern, Switzerland; ^2^Department of Emergency Medicine, Inselspital, University Hospital Bern and University of Bern, Bern, Switzerland

**Keywords:** head impulse test, learning curve, vertigo, emergency department, video head impulse test (vHIT)

## Abstract

**Objective:** The video head impulse test (vHIT) is nowadays a fast and objective method to measure vestibular function. However, its usability is controversial and often considered as a test performed by experts only. We sought to study the learning curve of novices and to document all possible mistakes and pitfalls in the process of learning.

**Methods:** In a prospective cohort observational study, we included 10 novices. We tested their ability to perform correctly horizontal head impulses recorded with vHIT. We assessed vHITs in 10 sessions with 20 impulses per session giving a video instruction after the first session (S1) and individual feedback from an expert for session 2 (S2) up to session 10 (S10). We compared VOR gain, the HIT acceptance rate by the device algorithm, mean head velocity, acceleration, excursion, and overshoot between sessions.

**Results:** A satisfying number of accepted HITs (80%) was reached after an experience of 160 vHITs. Mean head velocity between sessions was always in accepted limits. Head acceleration was too low at the beginning (S1) but improved significantly after the video instruction (*p* = 0.001). Mean head excursion and overshoot showed a significant improvement after 200 head impulses (*p* < 0.001 each).

**Conclusions:** We showed that novices can learn to perform head impulses invHIT very fast provided that they receive instructions and feedback from an experienced examiner. Video instructions alone were not sufficient. The most common pitfall was a low head acceleration.

## Introduction

The clinical head impulse test (cHIT) is a clinically convenient and efficient bedside test for the assessment of vestibular function in the high-frequency range, however, the correct execution and interpretation of this test needs training and experience (1). The head impulse test which was first described in 1988 (2), consists of a high acceleration head movement toward one direction (from eccentric to primary position) while the patient is fixating a target, usually the nose of the examiner (3). The examiner has to observe a so-called corrective saccade in patients with a deficient vestibulo-ocular reflex (VOR). Since the sensitivity and specificity of this test are low (3–5), it is recommended to use the video-oculography (VOG) device to record quantitatively HITs. This leads to a sensitivity and specificity comparable to the gold standard for quantitative head impulse testing, the search-coil-in-magnetic-field-technique. Such recorded video HITs, also known as vHITs, can be performed in the horizontal or vertical plane of the semicircular canals (6), however, current accuracy studies involved experienced neurootologist only and would not be generalizable to general or emergency physicians. A recent usability study from Heuberger et al. (1) demonstrated a learning curve of only three unexperienced examiners; however, this study was performed on a large number of different patients under non-controlled conditions.

We, therefore, sought to quantify the horizontal vHIT learning curve of novices without any cHIT or vHIT experience on a healthy subject under laboratory conditions and to compare different instructional methods. vHIT performance of novices was compared with the performance of an experienced neurootologist and with the performance of an automated, standardized HIT device.

## Materials and Methods

In this prospective cohort observational study, we recruited 10 medical students with no prior practical experience regarding cHIT and vHIT. We used a questionnaire to screen for practical and theoretical experience of the students. All students had a theoretical knowledge about HIT in general. All examiners performed 20 horizontal vHITs per session, 10 HITs in each direction. In total, participants performed 200 vHITs within 10 sessions. To quantify the impact of the different instruction methods (video instruction vs. expert instruction), there was no instruction given to the examiner prior to the first session except that the examiner had to stand behind the healthy subject while holding the head.

The vHIT device [EyeSeeCam (7, 8)] was already correctly calibrated by experienced research personal. The strap was very tight. Moreover, a good eye tracking was mandatory and adjusted by the research personnel. The right eye was tracked (monocular recording). All participants tested the same healthy subject with a normal VOR gain, normal vision, and a normal neck range of motion. The subject was asked to fixate on a light source at 1.5 m distance.

**Figure 1:**
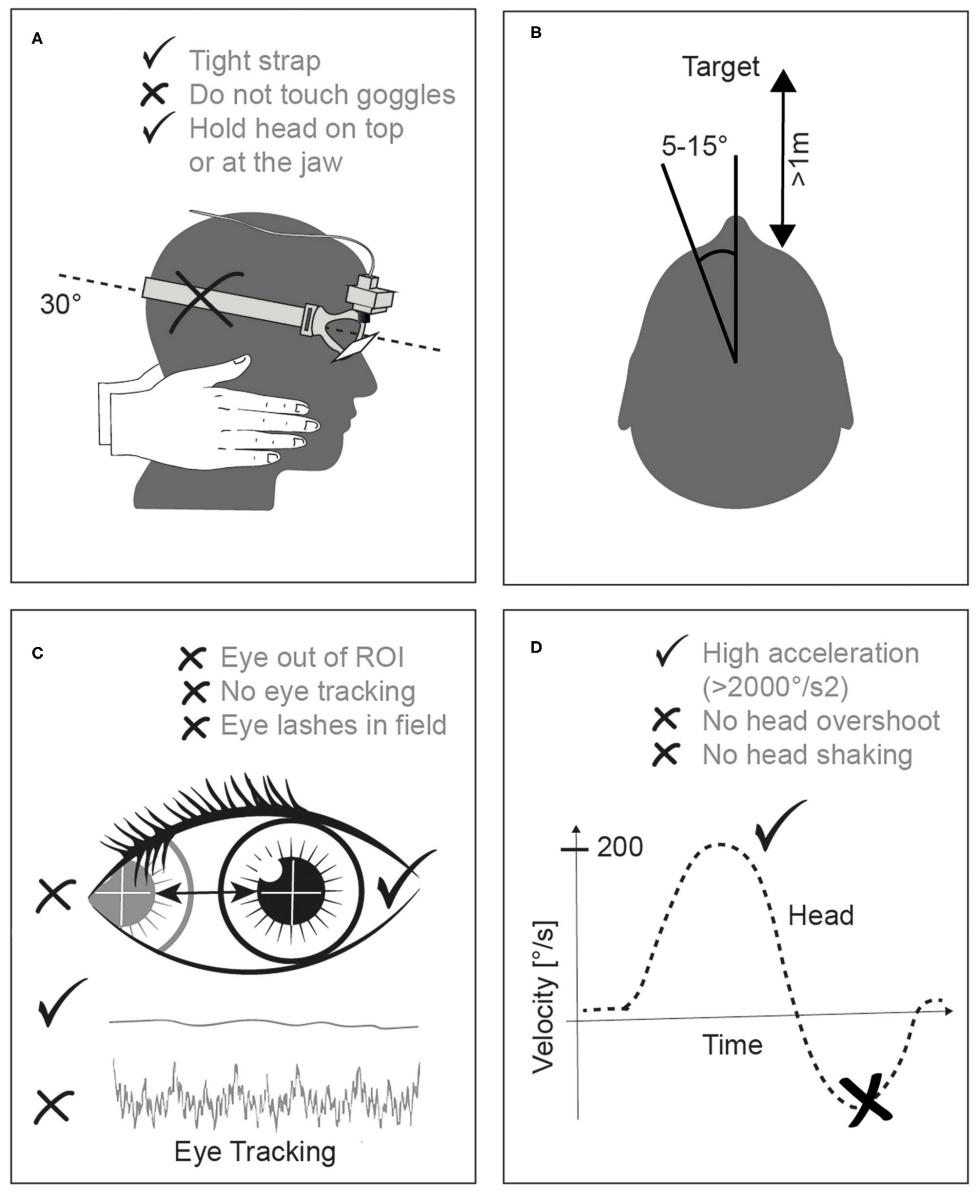
**(A)** shows the correct hand position avoiding touching the rubber strap of the goggles. A small angle of head excursion (**B**, 5–10°) and a distance of >1 m to the fixation point (avoiding vergence) is mandatory. The examiner has to make sure that the eyes are tracked correctly **(C)** avoiding any eye lashes being in the field of view. Finally, a minimal head acceleration of >2,000°/s^2^ **(D)** is necessary being in the correct dynamic range of the semicircular canal.

A video instruction provided by the vHIT company followed the first session (S1). The instructional video demonstrated optimal head impulses emphasizing a fast but small angled head movement, a sufficient distance from the fixation point, correct positioning of the examiner's hands, and the correct plane of head movements. The instructions also emphasized the need for small head excursions in order to keep the eye within the region of interest for the camera eye tracking. Figure 1 shows some key points, which have to be respected, and the common pitfalls. An experienced neurootologist gave corrective instructions after each session except after session one. vHIT performance of novices was compared with the performance of an experienced neurootologist and with the performance of an automated, standardized HIT device. This aHIT device consists of a servomotor assembled with a curved track guided by six bearings, which rotates a mouthpiece. The subject bites onto the mouthpiece covered with a silicon bite splint and as a result the head is moved horizontally in each direction according to predefined head velocity/acceleration profiles (Gaussian, peak angular velocity 150°/s, peak acceleration 3,000°/s^2^) (9).

The number of valid and accepted HITs were recorded for each side and session. Valid velocity thresholds ranged from 150 to 300 deg/s with a minimal acceleration of 1,830 deg/s^2^.

Primary endpoints were the number of valid (accepted) vHITs from the device per training session. Further endpoints were VOR gains measured at each session, head velocity, acceleration, excursion, and head overshoot. Raw head impulse data were processed and analyzed using a MatLab script (Matlab R2019b, Mathworks, Natick, Mass., USA). As a secondary endpoint, we categorized the number of mistakes. The effect of handedness and video instruction on the learning curve was also measured.

### Statistical Analysis

Differences in the outcome measures for head velocity, acceleration, excursion, and overshoot were estimated using separate linear mixed-effects models, with fixed effects for the session number (from 1 to 10), the side (categorical variable, i.e., left vs. right) and the instruction level (categorical variable, i.e., uninstructed vs. instructed by a video tutorial after session 1) as well as a subject-level random intercept to account for paired measurements. To investigate whether there were training-specific effects on the test direction, an interaction term between the variables side and session number was included. For the gain outcome variable, no data was available for session 1, as the subjects were not able to produce valid results without instructions. Therefore, the linear mixed effects model was the same as for the other outcomes measures, but the variable for instruction level was left out. A *P*-value of 0.05 was considered as statistically significant. All analysis performed in R environment [v3.4, R Core Team and the lmer package (10)] and descriptive statistics in SPSS (IBM Corp. Released 2017. IBM SPSS Statistics for Windows, Version 25.0. Armonk, NY: IBM Corp).

### Ethics

This study was approved by the local institutional review board (KEK-Nr 047/14) and a written consent form was obtained from the healthy subject.

## Results

**Figure 2:**
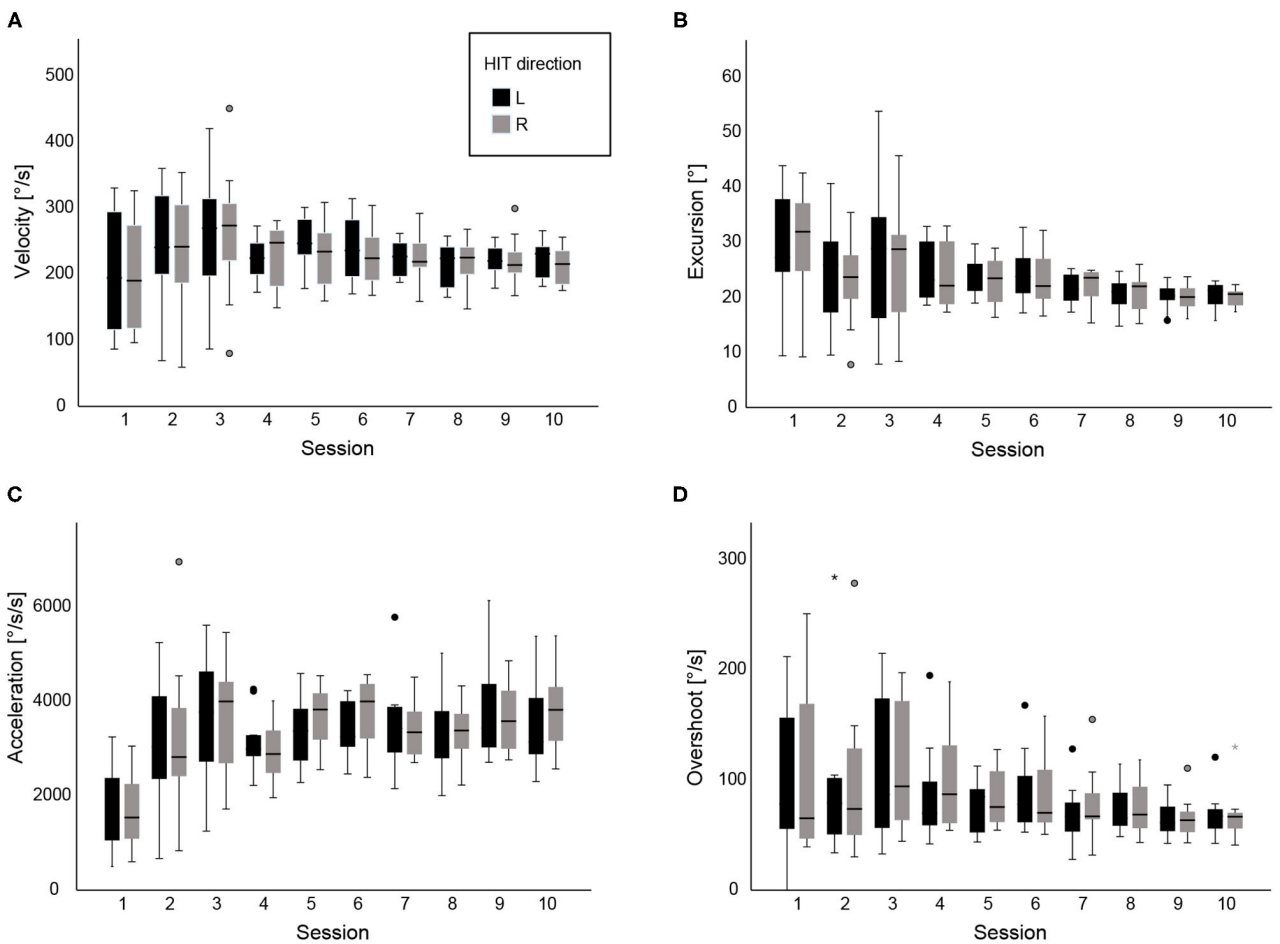
**(A)** shows box plots with head velocity (°/s) for each session, **(B)** median head excursion (°), **(C)** head acceleration (°/s^2^) and **(D)** head overshoot (head velocity toward the opposite direction, °/s) after the completion of 10 vHITs. Data are shown across all 10 sessions, grouped for left and right vHITs. Extreme outliers are marked with an asterisk (*) on the boxplot. Mild outliers are marked with a circle (⚬) on the boxplot.

We included 10 medical students, five males, and five females, aged between 22 and 32 (25.9, ± 2.7), and all of them right-handed. We present here data from 2,000 HITs collected after 100 sessions (20 HITs per session, 10 sessions per student) (Figure 2).

Peak head velocities were always within the acceptable range across sessions with a mean velocity at the beginning of 206°/s (±SD 90°/s). Velocity was significantly influenced during the learning process. After the video presentation, head velocity was significantly higher 241°/s (±SD 88°/s, *p* = 0.001). Overall, there was a minimal reduction of velocity across the sessions (−3.6° per session, *p* = 0.04), but velocity still remained within the accepted limits.

However, acceleration proved to be significantly influenced by video instruction: Mean acceleration before the instruction (S1) was 1,734°/s^2^ and reached a mean acceleration of 3,117°/s^2^ after the end of the second session, S2 (*p* < 0.001). On the contrary, no further significant improvement was shown after the second session across all following sessions (*p* = 0.2).

The angle of head movement (head displacement during vHIT) significantly changed after the video demonstration (*p* = 0.04) and continued to decrease significantly with personal instructions until the last session (−0.8° per session, *p* < 0.001). The mean excursion at the beginning was 29.7 (ranged from 9.2 to 43.8°) and decreased to 20.2° (ranged from 15.7 to 22.9°) at the last session.

In regard to head overshoot (turning the head immediately back to the midline, opposite direction), we observed statistically-significant difference only after personal instructions between the second and the last session (*p* < 0.001). Mean overshoot decreased from 103°/s (range 0−250°/s) to 68°/s (range 40−129°/s) in the end.

**Figure 3:**
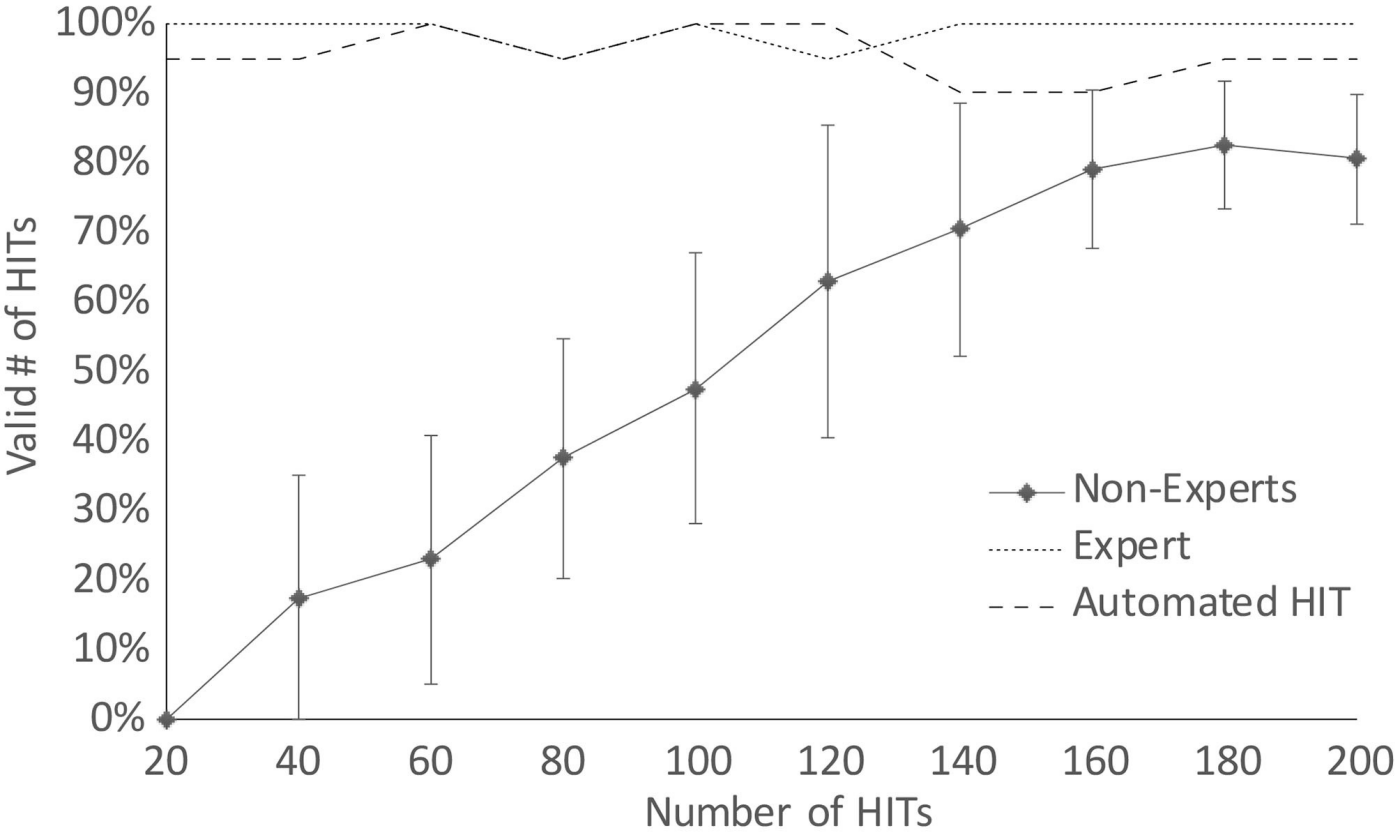
The mean percentage and standard deviations of valid, accepted vHITs for each session grouped by non-experts, expert and an automated vHIT.

Figure 3 shows the learning curve regarding the proportion of valid (accepted) vHITs for each session. We found that there was no significant difference between right and left accepted HITs. Also on average all students reached a maximum level of 83% valid HITs (−12.75) after 180 HITs, which is close to the performance of an expert or even with an automated, standardized head movement offered by the aHIT device.

**Figure 4:**
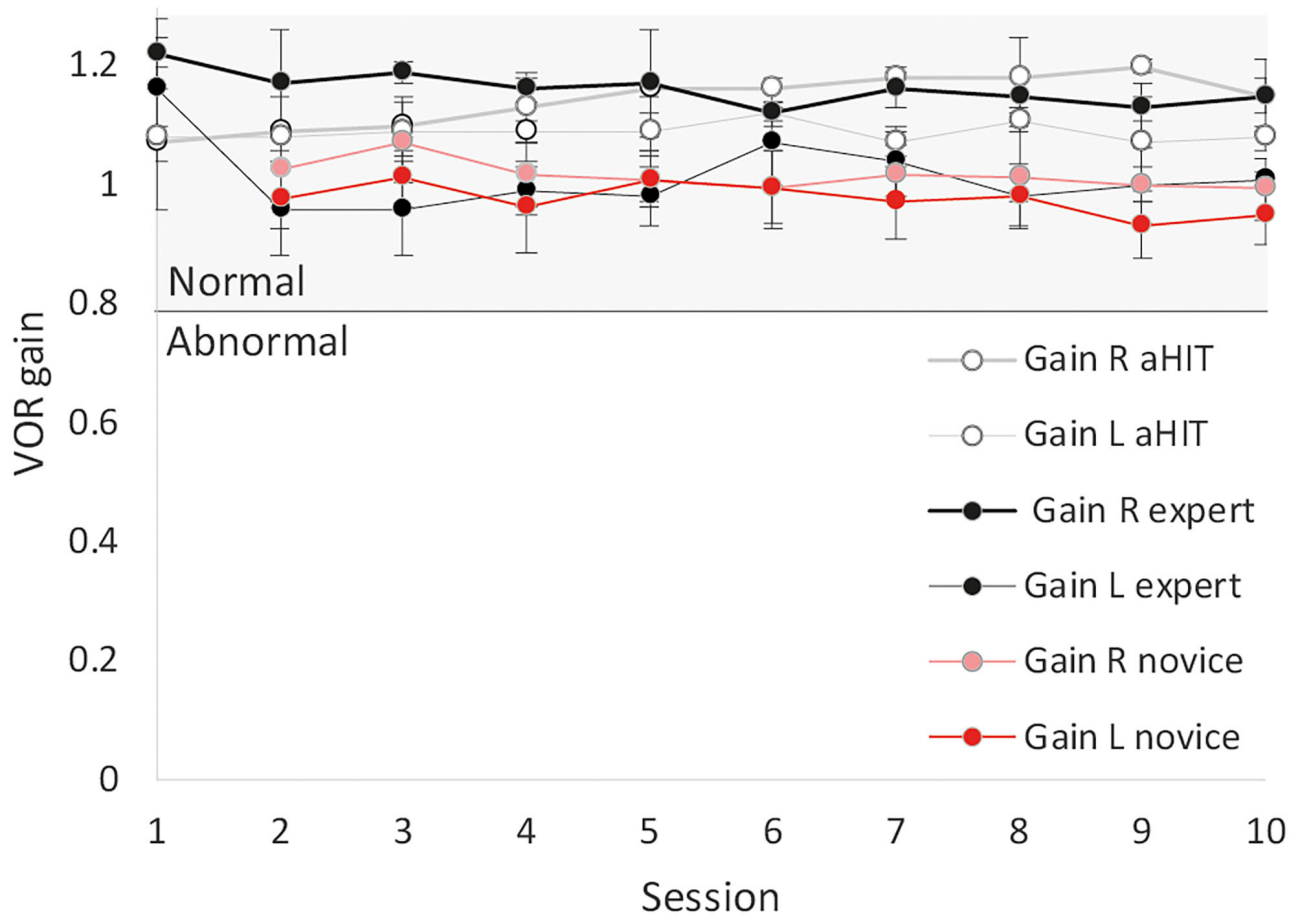
Mean VOR gains from valid HITs (see Figure 3) and confidence intervals for novices, an expert and the automated HIT (aHIT) device. VOR gain remained in the normal range across all sessions. Right VOR gain was slightly biased showing systematically higher gains than the left side due to the monocular recording of the right eye.

Averaged VOR gain did not statistically change across sessions (*p* > 0.05) although the total number of accepted HITs was increased after the first few sessions (Figure 4).

Table 1 shows the frequency of the most common mistakes the students made. Students never took advantage from the instant device feedback offered by the VOG algorithm but rather focused on the patient's head. Low accelerations and large head displacements were the most frequent reasons for invalid HITs and thus, HIT rejection by the device algorithm.

**Table 1:** Common mistakes and frequency after video instruction.

	**Common mistakes**	**Frequency [%]**
1	No use of device feedback	100
2	Low head acceleration	23
3	Big excursion of head movement	12
4	Touching the vHIT goggles	9
5	Head overshoot	8
6	Wrong hand position	6
7	Head shaking instead of impulse	4
8	Not random vHIT direction	2
9	Wrong head position (not in the canal plane)	0

## Discussion

Our study has shown that vHIT examination is feasible by non-experts after a short learning curve of 180 trials. The most important pitfalls leading to a vHIT recording failure were low head accelerations, large head overshoots, and extended excursion angles of head movement. Moving the head at high velocity (>150°/s) was not an issue as initially thought and VOR gain maintained in the normal range across all sessions. An instructional video improved head acceleration and reduced the head excursion size but did not prevent common mistakes such as head overshoot.

Some studies have shown that high velocity vHIT (240°/s) is superior to low velocity vHIT (80°/s) (11). Although it is fundamental to retain a high velocity during vHIT, we found that it was something that all the students achieved from the beginning and there was no improvement during the process of learning. However, this high velocity at the very beginning was achieved by expanding the head excursion angle and prolonging the duration of vHIT. Thus, a high head velocity was maintained by sacrificing head acceleration. There are four important points in regard to utility of a high head acceleration during vHIT: (1) The SCCs measure angular acceleration and there is a minimal amount of acceleration needed to move the endolymph and to cause cupula displacement due to the inertia of endolymph fluid. The dynamic response of the SCCs is therefore optimized for a higher frequency range (>0.05Hz). (2) The visual system is predominant at lower frequencies and optokinetic nystagmus serves as a backup system for angular VOR (aVOR), (3) The contralateral side is not driven to inhibition cut-off due to saturation effects and thus, the proportion of signal contribution from the contralateral side through commissural pathways is larger at low accelerations, and (4) VOR gains are smaller at higher accelerations (12), the position error between eye and head larger and thus, a larger corrective saccade will be needed. The biggest problem that almost all the students faced was the ability to reach a certain point of acceleration >2,000°/s^2^, which is the most common issue but our results have shown that the video demonstration was very effective.

Some of the limitations of eye-tracking during HIT could be big eyelashes covering the pupil, makeup reflecting the infrared light, or large head excursion leading the ROI outside the camera view. A large head excursion should also be avoided because of a possible neck trauma especially in patients with a previous history.

Head overshoot has been associated with significantly higher vHIT velocity and duration, lower slow phase amplitude of the impulses, and consequent higher saccades' latency and lower saccades amplitude (13). Head shaking and overshoot would have an impact on test results in patients by inducing wrong direction saccades Covert-Anti-Compensatory Quick Eye Movements (CAQEM). Such saccades are believed to be either the result of gain asymmetry (14) or the result of testing the opposite ear. Our study has shown that vHIT misapplication with head overshoot needs a hands-on practice as well in order to be reduced.

VOR gain results after each session still remained in the normal range of ~1, however, the calculation of a mean VOR gain, which is based on a too small sample size (due to a small number of accepted HITs), might be prone to wrong gain estimations. A recent study showed, that VOR gain was meaningful and stable even in traces with artifacts, provided that a minimal number of 10–20 vHITs per session was performed (15).

A goggle slippage leads to artifacts like head overshoot so that the device sometimes rejects the vHIT or makes a false gain calculation (16). One potential source of goggle slippage is an insufficient fitting of the goggles; however, we applied all vHITs on the same subject with a very tightened strap, so that the students did not have to manage this problem.

A wrong hand position can also move the skin leading to indirect camera vibrations (Figure 1A). Touching the vHIT goggles is a common mistake that almost all examiners do, even the experienced ones (17) which also leads to artifacts.

Active and passive HITs render different VOR gain results due to planned, predicted head movements (18). For passive vHITs, however, the examiner has to avoid any predictive cues such as anticipation of the planned head direction since prediction during passive vHIT has an impact on the gain of VOR (19, 20). Some students forgot to follow the instructions of the video regarding the randomness of cadence and HIT direction. We also observed a decent asymmetrical gain between right and left impulses due to the goggles geometry and camera position on the right eye (21).

The orientation of horizontal SCC is ~30° from the horizontal plane or ~20° upward from Reid‘s baseline, a plane that connects the bony portion of the external auditory canal to the floor of the bony rim of the orbit (22). Therefore, the optimal head position, in order to test the lateral (horizontal) SCC plane is head pitching 30° down (Figure 1A) (23). Our subjects met no problem with that. We tested, however, only horizontal head impulses and not impulses in the vertical plane, which is much harder to learn and to perform.

Another bias could be the position of the examiner in relation to the position of the patient. There are predominately two techniques, the sitting in front or standing in back of the patient technique. Sitting in front of the patient as done clinically can influence the outcome of left and right vHITs because the examiner has to sit opposite to the patient, but diagonally or slightly displaced: The patient must still be able to see the fixation target on the wall by watching over the shoulders of the examiner. Using the examiner's nose, as a fixation target does not apply in vHIT in order to avoid any effects from vergence. Sitting in front of the patient, but asymmetrically displaced, might therefore lead to an asymmetrical magnitude of acceleration and excursion angle. In addition, the sitting in front technique is more prone to artifacts (15). Our statistics have shown that standing from behind made no difference between right and left HITs. The sitting in front technique, however, might still be an option for physicians applying vHIT at the bedside, where standing behind the bed would not be impossible.

Our study had some limitations. We applied vHIT on one healthy subject in order to avoid any inter-subject variability regarding goggles fitting, neck stiffness and eye tracking. However, results might not be fully generalizable to other subjects because of anatomical variations (facial relief, eye lashes, pupil size etc.) with impact on goggles fitting and eye tracking. The performance of vHIT is inevitably more difficult if somebody tries it on patients. A patient could have spontaneous nystagmus biasing the eye tracker. Acute dizzy patients tend to close their eyes. They suffer from nausea so that their attention and cooperation are not ideal. We assessed only the performance of head movements. Since there are many brands on the market, we did not want to test software usability, which might be different for each device but also for each device generation. The vHIT acceptance rate by each device algorithm might be different. Velocity thresholds for valid vHIT are customizable and might be adjusted by the software user. We included only medical students and did not assess other professionals, such as nurses or medical assistants. We still think that our results could be generalizable because students had no previous knowledge and no special skills. That could also be a subject for a future study. One concern is the sustainability of the learned skills. We have not tested whether the students were still able to perform a vHIT after a period of some months with the same performance as before. Future studies should include an assessment of various medical professionals, different devices and long term sustainability results.

### Future Implications for vHIT

Further studies are mandatory before implementing vHIT in the ED since we did not test the ease of device use including putting the goggles correctly on the subject, setting the focus of the camera, defining the right distance between the wall and the subject, operating the device software, and the calibration process. This study, however, is a first step toward a point-of-care vHIT testing in the ED. The concept of widespread use of vHIT as an “eye ECG” in analogy to the use of ECG in the ED, was already propagated by Newman-Toker et al. (24). ECG was historically performed by cardiologists only and now it is part of a routine assessment in EDs performed by trained nurses or technicians. In addition, vHIT could also be used as a training tool for nurses and emergency department staff. The vHIT software gives an instant feedback about the validity of HITs and allows the conduction of standardized and reliable vHIT recordings. These recordings in the future can be sent to an expert for an assessment through a computer. A widespread use of vHIT in the ED could offer a more accurate, timely, and efficient ED diagnosis in the future.

## Conclusions

Our results suggest that conducting an accurate head impulse recorded with vHIT is not limited to experts. Although many potential errors can happen, we showed that non-experienced users can learn to move the subject's head in the correct plane, velocity and acceleration with an optimal excursion size provided that they receive instructions and feedback from an experienced examiner. Video instructions alone were not sufficient. The most common pitfall was a very low head acceleration.

## Data Availability Statement

The original contributions presented in the study are included in the article/supplementary material, further inquiries can be directed to the corresponding author/s.

## Ethics Statement

The studies involving human participants were reviewed and approved by Kantonale Ethikkommission Bern (KEK). The participants provided their written informed consent to participate in this study.

## Author Contributions

AK: major role in the acquisition of data interpreted the data and drafted the manuscript for intellectual content. TS: interpreted the data and revised the manuscript for intellectual content. MC: revised the manuscript for intellectual content. GM: design and conceptualized study, analyzed the data, major role in the acquisition of data interpreted the data, and revised the manuscript for intellectual content. All authors: contributed to the article and approved the submitted version.

## Conflict of Interest

The authors declare that the research was conducted in the absence of any commercial or financial relationships that could be construed as a potential conflict of interest.
